# Scalp Reconstruction: A Review of the Literature and a Unique Case of Total Craniectomy in an Adult With Osteomyelitis of the Skull

**Published:** 2014-07-19

**Authors:** John P. Tutela, Jonathan C. Banta, Travis G. Boyd, Sean S. Kelishadi, Saeed Chowdhry, Jarrod A. Little

**Affiliations:** ^a^Division of Plastic and Reconstructive Surgery, University of Louisville, Louisville, Ky; ^b^School of Medicine, University of Louisville, Louisville, Ky; ^c^Department of Otolaryngology, University of Louisville, Louisville, Ky

**Keywords:** skull osteomyelitis, total craniectomy, omental free flap, scalp reconstruction, cranium

## Abstract

**Objective:** Osteomyelitis of the skull is a rare condition that can lead to systemic illness, bone loss, intracranial complications, and mortality. Osteomyelitis of the skull typically presents as the boney invasion of an overlying infection of the scalp or sinuses, and it is typically treated with antibiotics and proper wound care. Surgical debridement of the affected bone in the form of a craniectomy may be initiated to stop the progression of the infection when antibiotics fail and the underlying bone becomes grossly eroded. **Method:** The authors present the case of a 54-year-old woman who required a total craniectomy after developing full-thickness osteomyelitis. A free omental flap along with dermal grafts and split-thickness skin grafts were utilized for soft tissue coverage. A semi-rigid helmet was used to provide durable protection to the brain. **Results:** Omental free flap with skin graft coverage provided this patient with durable and long-term soft tissue coverage for a total craniectomy defect, as well as the ability to regain mental status. **Conclusions:** Many factors must be analyzed when approaching composite defects of the scalp. Modality of treatment must be customized to the individual, and the decisions should be based on whether the defect is composed of soft tissue, bone or both, its size, etiology, and presence of a cerebral spinal fluid leak. The goals of treatment are restoration of durable soft tissue coverage, protection of vital underlying structures and control of cerebral spinal fluid leaks.

Osteomyelitis of the skull is a rare but well-studied condition that can lead to systemic illness, bone loss, intracranial complications, and mortality. It usually presents as a chronic process that arises from an overlying infection of scalp. Osteomyelitis of the skull has also been documented as developing from pediatric tumors and specific infections such as aspergillus, cryptococcus, blastomycosis, mucormycosis, salmonella, mycobacterium, propionibacterium acnes, treponema pallidum, tuberculosis, varicella, and human immunodeficiency virus (HIV).^1^^,^^2^ Furthermore, skull osteomyelitis has recently gained importance due to its association with immune-depressed states such as HIV, prolonged neutropenia in critically ill patients, drug-induced immune suppression in organ transplantation, and chemotherapy treatment for solid tumors.^1^ Treating osteomyelitis of the skull has benefited from the use of antibiotics; however, the debridement of necrotic bone still remains a viable treatment should medicine fail. In such cases that warrant surgery, specific scalp reconstructive techniques, such as primary closure, skin grafts, tissue expansion, local and regional flaps, and free tissue transfer, exist to provide durable soft tissue coverage, protection of underlying structures, and control of cerebral spinal fluid leaks.^3^

The authors present the case of a 54-year-old woman who required a total craniectomy after developing full-thickness osteomyelitis of her entire cranium due to the progression of a scalp wound sustained less than 1 year prior. To the best of the authors’ knowledge, this is the only case of skull osteomyelitis in the English literature that necessitated a total craniectomy. The success of this course of treatment sheds light on the extent of removal of necrotic bone that can be taken when treating widespread skull osteomyelitis in patients that fail conventional treatment with antibiotics.

## METHODS

Our patient, VS, is a 54-year-old white woman who sustained a scalp wound while moving a refrigerator at work. She developed a hematoma that was observed and subsequently became infected. Her medical history was insignificant except an extensive tobacco use. She underwent an incision and drainage; however, her scalp infection was recalcitrant to treatment and required multiple debridements. Eight months after her initial injury, she was found unresponsive by her family at home and was brought to an outside hospital. Computed tomographic (CT) scan of her head demonstrated a right parietal lobe subdural empyema causing a left midline shift and a moth eaten appearance of the calvarium. Her white blood cell count was 28,000. She underwent a right frontoparietal craniotomy. On her first postoperative day, she was taken back to the operating room (OR) for a decompressive craniectomy of the right frontotemporal parietal bone for increasing intracranial pressure.

**Figure 1:**
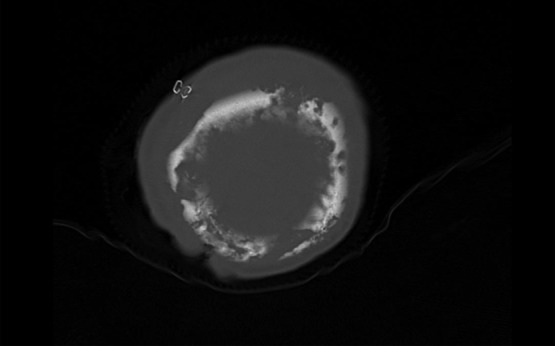
Computed tomographic image demonstrating full-thickness osteomyelitis of the cranium.

**Figure 2:**
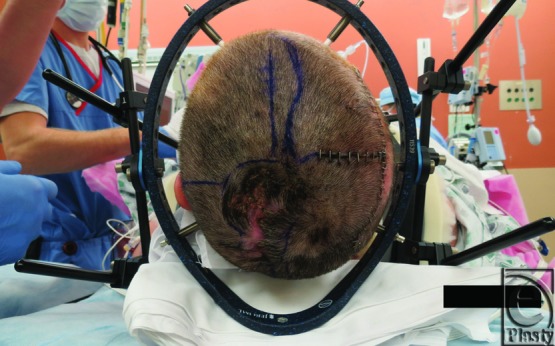
Preoperative photograph showing an 8 × 8 cm^2^ area of a chronically infected scalp wound. A halo was secured around the patient's head to suspend it without pressure in the postoperative period.

The patient's initial culture of the drained subdural empyema grew *Streptococcus intermedius* and the patient was started on ceftriaxone. Upon stabilization on hospital day 7, she was transferred to our institution for further management. Her CT scan on her admission to our institution confirmed full-thickness destruction of her cranial vault (Fig 1). On presentation, she was verbally nonresponsive, would withdraw from pain, and displayed left-sided weakness. Upon examination, there was an 8 × 8 cm2 area of chronically infected scalp (Fig 2). Her white blood cell count was 23,000 and her alkaline phosphatase was 389.

**Figure 3:**
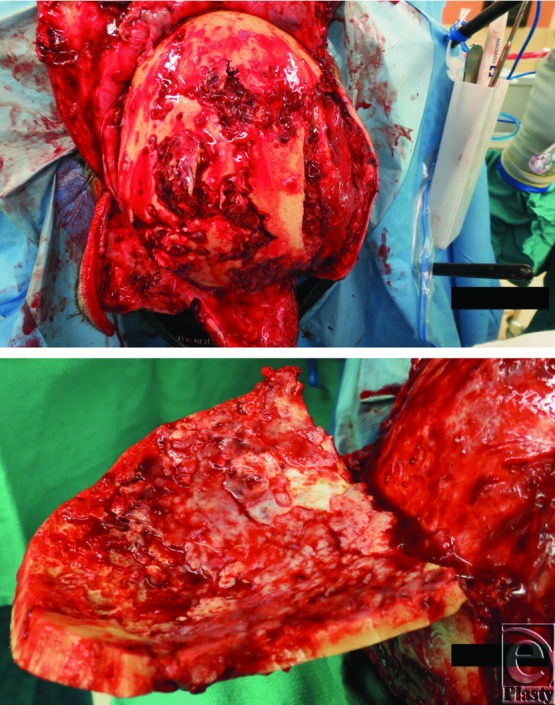
Intraoperative photographs showing full-thickness osteomyelitis of the cranium as well as the “moth-eaten” appearance of the underlying bone after the bone flap was lifted.

**Figure 4:**
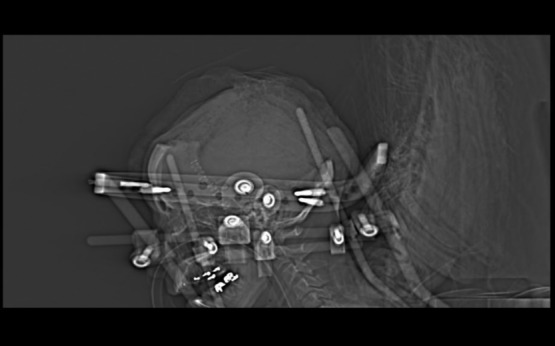
Sagittal computed tomography of patient's head showing the extent of skull debridement required for treatment.

The patient was taken to the OR, in conjunction with the neurosurgical team. A halo was placed to keep her head suspended without pressure on her bed postoperatively. Scalp flaps were raised revealing full-thickness destruction of the cranium (Fig 3). Debridement of the infected cranium resulted in a skull defect approximately 50 cm in circumference (Fig 4). A dural defect was noted at the site of the original craniectomy site and was covered with Duragen (Integra LifeSciences Corporation, Plainsboro, New Jersey). Culture from bone specimen grew *Pseudomonas aeruginosa*, and her antibiotic was changed to cefepime. Wet to moist dressings with one-eighth strength daikins solution were then placed under the scalp flaps and the flaps were approximated. Dressings were changed every 8 hours.

On postoperative day 7, she was taken back to the OR for further debridement and for drainage of a frontal lobe abscess. Following debridement of devitalized dura, bovine pericardium (Edwards Lifesciences Corporation, Irving, California) was used as a sling to support the brain parenchyma. At this time, cultures from the wound grew *Candida albicans*, but the abscess failed to show any growth. Amphotericin B was added to her antimicrobial regimen.

**Figure 5:**
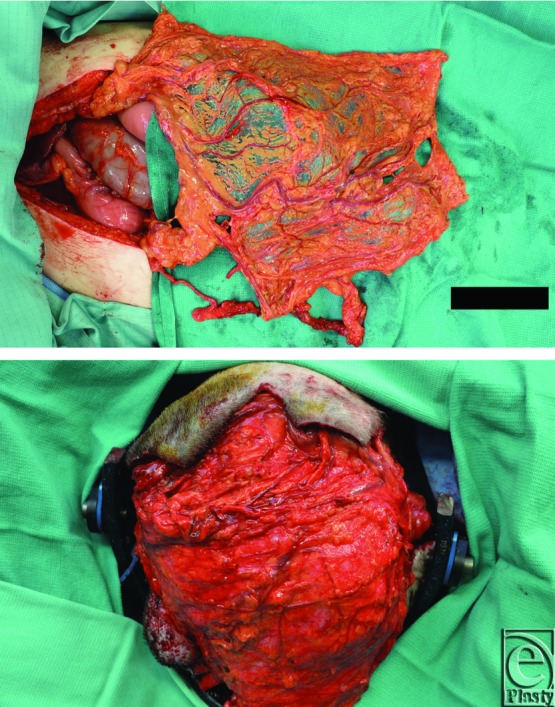
Intraoperative photographs showcasing free omental flap harvest and subsequent coverage.

**Figure 6:**
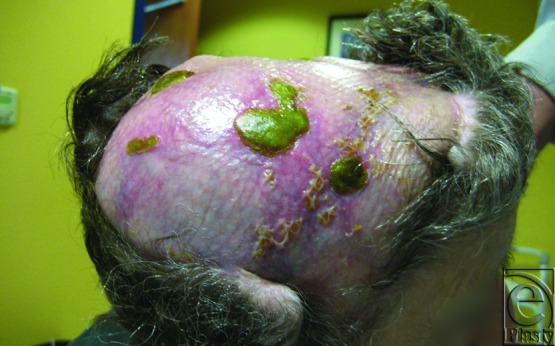
Postoperative photograph of the patient's scalp showing the skin graft overlying the defect.

On postoperative day 27, when the wound cultures showed no growth from qualitative cultures, the patient was taken to the OR for wound coverage with a free omental flap (Fig 5). A free latissimus dorsi flap was also high on our list of potential donor flaps for its size and durability, however, because our patient lacked proper cranial support of her brain to allow for lateral decubitis or prone positioning we felt it was best to keep her in the supine position. Omental harvest allowed for this approach. Acellular dermal matrix (Alloderm, Lifecell Corporation, Bridgewater, New Jersey) was secured to the granulating bed overlying the dura and under the remaining scalp flaps with the intention of adding durability to the soft tissue reconstruction. The omental flap was harvested, anastomosed to the patient's right superficial temporal artery, and laid over the newly placed acellular dermal matrix. A split-thickness skin graft was used for final coverage over the omental flap once the viability of the flap was verified (Fig 6).

## RESULTS

Throughout the patient's hospital stay, the patient recovered from her left-sided weakness and started responding appropriately to questions. Furthermore, with continued physical rehabilitation, she regained full mobility. As the patient recurred a yeast infection of her remaining scalp flap, synthetic cranioplasty was deferred and protection was provided by a helmet. At a 2 month follow-up, she remained free of infection. Omental free flap with dermal and split-thickness skin grafts provided durable long-term soft tissue coverage for a total craniectomy defect caused by osteomyelitis of the skull in an adult.

## DISCUSSION

The treatment for osteomyelitis of the skull has been extensively reviewed in the literature due to the heightened risk of the neurological complications that are inherent in any infection that threatens invasion into the central nervous system. Over the past half century, osteomyelitis of the skull has seen an increase in occurrences despite the widespread use of antibiotics, due mainly to the rise of immunocompromised patients undergoing organ transplantation, treatment for HIV, chemotherapy for solid tumors, and prolonged stays in intensive care units.^1^ Laboratory values that are typically elevated in cases of osteomyelitis are blood leukocyte level, erythrocyte sedimentation rate, and C-reactive protein. However histopathological and micropathological examination of bone remain the gold standard for diagnosing osteomyelitis.^1^ With the advent of CT, physicians gained new insight into the boney erosion and the damage of osteomyelitis—a profound advancement from the x-rays of the past. Magnetic resonance imaging proves to be very sensitive in detecting osteomyelitis in the acute stage and provides a good view of the abnormal marrow edema in the first few days of the infection.^4^ Today, magnetic resonance imaging is considered to be the best way to demonstrate the extent of cortical destruction found in osteomyelitis.^5^ Radiographs fail in their ability to monitor osteomyelitis therapy because of the extensive time it takes for the bone to appear normal after a successful treatment.^6^ However, use of bone scintigraphy using technetium 99 and gallium citrate scans, as well as positron emission tomography, have been used with great success to monitor a patient's therapeutic response to treatment.^7^ The routine checking of C-reactive protein and/or erythrocyte sedimentation rate can be implemented throughout the course of treatment to gauge the effectiveness of the osteomyelitis treatment. If these serum inflammatory markers fail to return to normal levels, further diagnostic testing can be implemented or the treatment can be modified.^8^ To this day, the preferential treatment of osteomyelitis of the skull is often debridement of diseased bone, and 4 to 6 weeks of intravenous antibiotics.^1^ When dealing with comorbidities such as peripheral vascular disease, diabetes, and radiation, the treatment for osteomyelitis can be more challenging due to the impaired wound healing brought about by such conditions.^9^ Until this case, there has never been a report of a patient requiring a total craniectomy as a result of skull osteomyelitis. In fact, a total craniectomy is a very rare procedure and is only routinely used in the treatment of craniosynostosis in infants.^10^

Reconstructive options for scalp and cranial defects have a wide range of options from primary closure for small defects to free tissue transfer for defects that are larger and more complex.^3^

Allowing minor scalp wounds to heal by secondary intention is the simplest form of treatment if the patient is willing to accept alopecia. When a scalp defect is only a few centimeters in diameter, primary closure is the acceptable form of treatment. In this instance, wide undermining and galeal scoring aide in advancement and decrease the tension on the final wound closure. When a scalp defect is too large for primary closure, a primary split-thickness skin graft can be used as long as there is a vascularized bed underneath the defect that will allow the graft to “take.”^11^ This process also has an inherent disadvantage of alopecia, as well as mismatched contour, color, and texture when compared to the surrounding scalp. To avoid this discrepancy in appearance, tissue expansion of the surrounding scalp can be used to allow the surgeon to replace like tissue with like tissue. For this method, an expandable silicon device is placed under the adjacent uninjured scalp and either inflated acutely to help cover smaller defects, or chronically (2-3 months) if the defect requires a greater amount of tissue for coverage.^12^ Unfortunately, this method of reconstructive cranial surgery has a complication rate as high as 39% which includes hematoma, infection, implant exposure, alopecia, wide scar, and cranial vault deformation.^13^

Both local and regional flaps are reserved for medium to large defects of the scalp and provide the surgeon with the largest variety of scalp reconstruction techniques. Local flaps consist of transposition, advancement, and rotation flaps. Each type of local flap comes with its unique indication, advantage, and disadvantage, and the implementation of whichever local flap usually depends on the surgeon's preference and unique nature of the patient's scalp defect. These unique local flaps have the ability to cover up to 50% of exposed bone including the calvaria and dura. For defects in the frontal, temporal, or occipital areas, a single local axial flap may be used based off the temporal or occipital artery. For defects of the central calvarium, multiple rotation or transposition flaps can be used with great effect. Local flaps have the advantage of good blood supply, minimal donor site morbidity, and of providing good cosmetic results. However, care must be given when harvesting local flaps due to the possibility of damaging the overlying hair follicles or frontal branch of the facial nerve.^14^ When there are hostile wound conditions surrounding the primary scalp defect due to trauma, osteomyelitis, osteoradionecrosis, and removal of malignant tumors, a regional flap provides a suitable alternative to a local flap.^15^ Furthermore, the poor elasticity of the scalp may warrant the use of regional flaps when it proves too difficult for the surgeon to use a local flap.^16^^,^^17^ The most commonly used regional flaps are the trapezius, latissimus dorsi, pectoralis major, and splenius capitus flaps. One benefit of regional flaps is that they bring remote tissue that is out of the zone of pathology to the defect.

The use of free tissue transfer flaps has generally been reserved for larger lesions with an unfavorable wound environment such as heavy trauma, osteomyelitis, osteoradionecrosis, radiation, or previous flap failure.^14^ Free flaps that have been described in literature for use in reconstructive surgery of the cranium to include radial forearm, latissimus dorsi, rectus abdominis, omentum, scapular, and Scarpa.^15^^,^^18^^-^20 In their publication, Beasley et al proposed a treatment and staging algorithm for the use of free flaps on lesions greater than 50 cm^2^ on the forehead and greater than 200 cm^2^ on the scalp. Furthermore, Beasley et al state that free flap reconstruction tended to result in better wound healing and a shorter hospital stay due in large part to the excellent vascularity of the free flaps. In their case series and review of literature, Seitz et al state their belief that composite tissue transfer including vascularized bone is superior to soft tissue alone or soft issue with prosthetic cranial replacement when applicable, as it can successfully cover large complex cranial defects, and restore contour and skeletal support. As a result, they felt they improved functional outcome with limited donor site morbidity when treating defects of the scalp.

In terms of an omental free flap for use in reconstruction of complex scalp defects, Losken et al showcase the versatility and high success rate of omental free flaps. The omentum is pliable and has the ability to fill the irregular borders that are sometimes present in scalp reconstruction procedures. Furthermore, patients who receive an omental free flap are better equipped to fight infections at the wound site due to the abundant vascularity and unique immunologic properties of the omentum. The omental free flap has also proven to be a good alternative to the traditional free flaps when they are either too hard to harvest or not big enough to cover the defect. One unique disadvantages of using omental free flaps is the need to enter the abdominal cavity. Intra-abdominal complications of omental free flap harvest include volvulus, intestinal obstruction, or enterotomies, and special caution should be taken when the patient has peritonitis or adhesions due to prior intra-abdominal surgeries.^21^

## CONCLUSION

We report the unique case of a 54-year-old woman who required a total craniectomy after developing full-thickness osteomyelitis of the entire cranium due to a scalp lesion obtained less than 1-year prior. Although surgical debridement of necrotic bone and the use of antibiotics is a well-documented treatment for skull osteomyelitis, the large extent of this particular infection dictated an equally large debridement. Cranial reconstruction using an omental free flap with split-thickness skin grafts was successful in providing soft tissue coverage while a helmet provided rigid protection. The patient regained preinjury neurologic function and remains free of infection.

Skull osteomyelitis is a rare and potentially fatal affliction that necessitates prompt diagnosis and treatment with surgical debridement and antibiotics. Early suspicion and recognition of the specific organism causing this treatable condition is key to the patient's prognosis. The goals of treatment are control of infection restoration of durable soft tissue coverage, protection of vital underlying structures, and control of cerebral spinal fluid leaks.
